# Supplementary material for: Exploring aggregation genes in a P. aeruginosa chronic infection model
Source: J Bacteriol. 2024 Dec 11;207(1):e00429-24. doi: 10.1128/jb.00429-24 (PMC11784459; doi:10.1128/jb.00429-24)
Supplement: Supplemental materials — Fig. S1 to S5, Table S2, and figure legends. [file jb.00429-24-s0001.pdf]

Supplementary figure legends

Table S1 – The most dysregulated differentially expressed genes from aggregate sample, comprised of 13 ncRNAs and 50 coding genes. Sheet 1 shows genes ordered by the condition in which they were identified, Sheet 2 shows genes ordered by fold change. 3 biological replicates, significance cutoffs 2-fold change, FDR 0.05. When grouped by condition, sf = synthetic CF sputum media (SCFM2) lb = LB broth, pm = plus mucin, mm = minus mucin.

Table S2 – Differentially expressed pyocins in aggregates. Highlighted pyocins are found in both mucin-containing conditions.

Figure S1 – Genes in common and phenotype biomass over time. Genes specific to CF-like environment are needed for WT aggregation. (a) Venn diagram showing number of unique and overlapping genes between several *in vitro* models and *ex vivo* samples. (b) Total biomass over time of transposon mutants grown in SCFM2. Entropic mutants maintain large populations with rapid doubling times, while impaired mutants have a comparable or slower than WT growth rate.

Figure S2 - Complementation of *Pa* Tn-mutants in synthetic CF sputum media (SCFM2). (a) comparison of Tn-mutant (clear bars) and complement (lined bars) after 4 hours growth (b) after 7 hours growth. (c) comparison of WT PAO1 and complemented *Pa* mutants after 4 hours growth (d) after 7 hours growth. Data represents 3 biological replicates, * denotes statistical significance and ns, no significance as determined by two-way ANOVA with Fischer's LSD multiple comparisons (a, b) or Kruskal Wallis (c, d) ($p < 0.05$).

Figure S3 – Prediction pipeline. This pipeline uses sequential and structural homology as a basis to predict the function and pathway of hypothetical proteins important in aggregates. AlphaFold is used to model protein complexes, and STRING is used to predict interactions with other proteins.

Figure S4 – WT co-culture. Comparisons of mutant and (a) WT total biomass, (b) average aggregate size, and (c) number of planktonic cells at 5, 10, and 15 hours. Mutants are grouped by phenotype. 3 biological replicates +/- SEM, significance of mutants vs. WT calculated using ordinary two-way ANOVA (P value <0.0001) with Fischer's LSD multiple comparisons test (alpha 0.05).

Figure S5 - Comparison of structural similarity of PA5102 and two *pseudomonas* proteins that have similar functions to the function predicted by our prediction pipeline. (a) The AlphaFold structure of PA5102. (b) PA5102, shown in pink, is compared to *Pseudomonas aeruginosa* fatty acid desaturase DesA, shown in green. DesA is associated with membrane-bound fatty acids and has a root mean square deviation (RMSD) score of 15.01 Å when compared to PA5102. (c) PA5102, shown in pink, is compared to *Pseudomonas aeruginosa* fatty acid desaturase DesB, shown in blue. DesB is associated with exogenous fatty acids and has a RMSD score of 11.561 Å when compared to PA5102, demonstrating that it is more structurally similar to PA5102 than DesA.

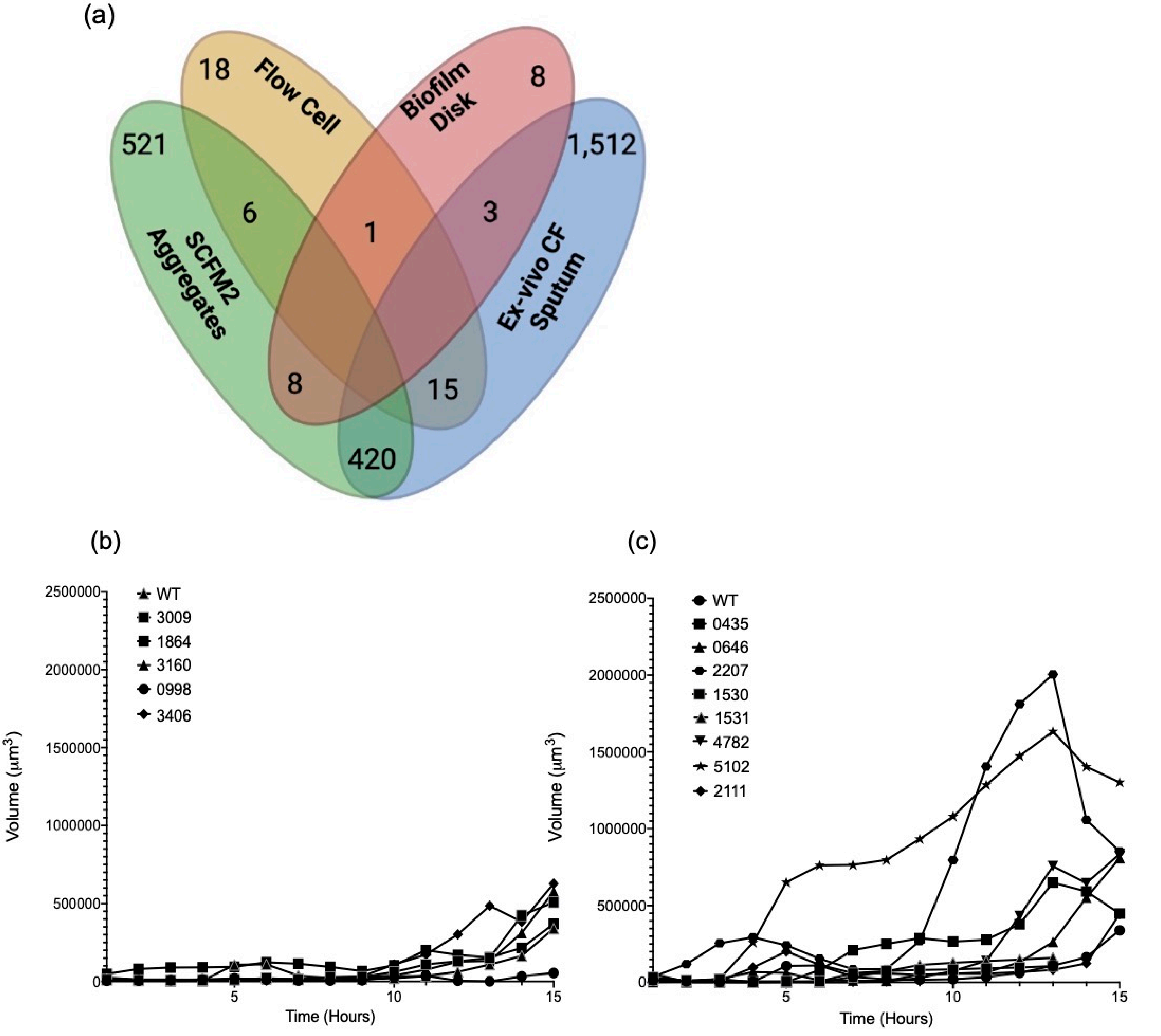

(a)
18
Flow Cell
Biofilm Disk
8
521
1,512
6
1
3
SCFM2 Aggregates
Ex-vivo CF Sputum
8
15
420
(b)
WT
3009
1864
3160
0998
3406
Volume (μm³)
Time (Hours)
(c)
WT
0435
0646
2207
1530
1531
4782
5102
2111
Volume (μm³)
Time (Hours)

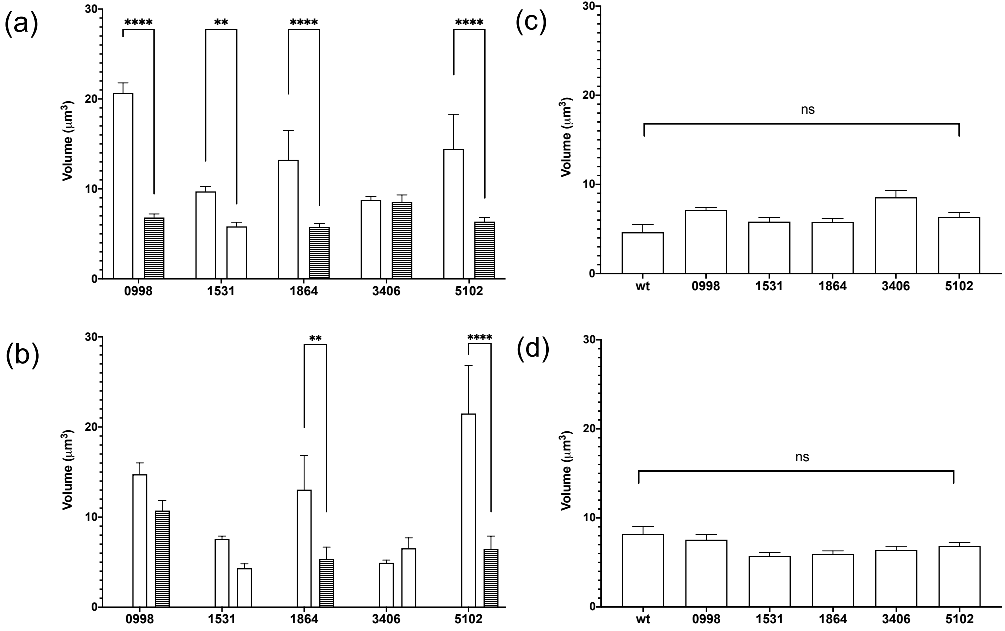
(a)
(b)
(c)
(d)
Volume (μm³)
0998
1531
1864
3406
5102
wt
****
**
ns
0
10
20
30

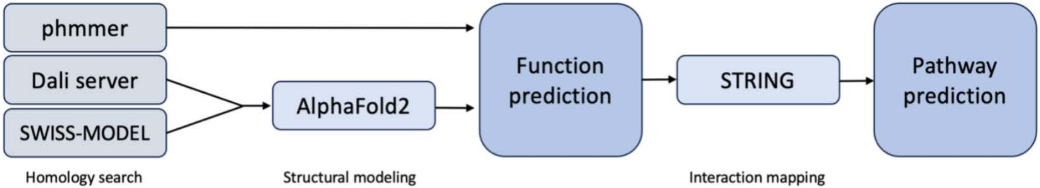
phmmer
Dali server
SWISS-MODEL
AlphaFold2
Function prediction
STRING
Pathway prediction
Homology search
Structural modeling
Interaction mapping

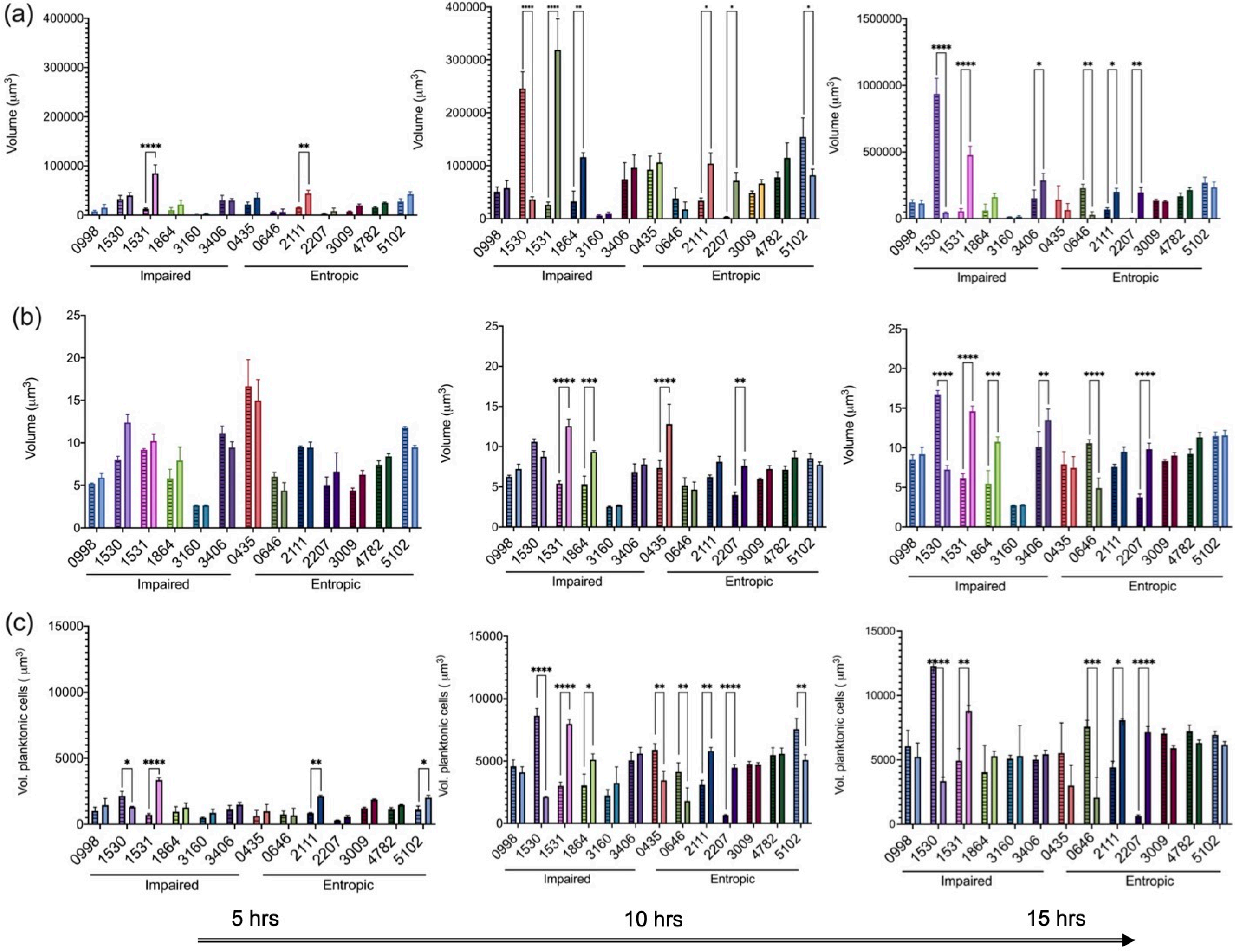

(a)
(b)
(c)
Volume (μm3)
Vol. planktonic cells (μm3)
0998
1530
1531
1864
3160
3406
0435
0646
2111
2207
3009
4782
5102
Impaired
Entropic
5 hrs
10 hrs
15 hrs

Downregulated Pyocins	
**SCFM+m**	**LB+m**
PA0616 - R	PA0615- R
PA0621 - R	PA0618 - R
PA0627 - R	PA0620 -R
PA0640 - F	PA0623 - R
PA0643 - F	PA0624 - R
PA0644 - F	PA0626 - R
PA0645 - F	PA0628 - R
PA0646 - F	PA0629 - Lysis
PA0647 - F	PA0630 - Lysis
PA0648 - F	PA0633 - F
pyoS5 - S	PA0636 - F
	PA0637 - F
	PA0639 - F
	PA0640 - F
	PA0641 - F
	PA0643 - F
11 Total	16 Total

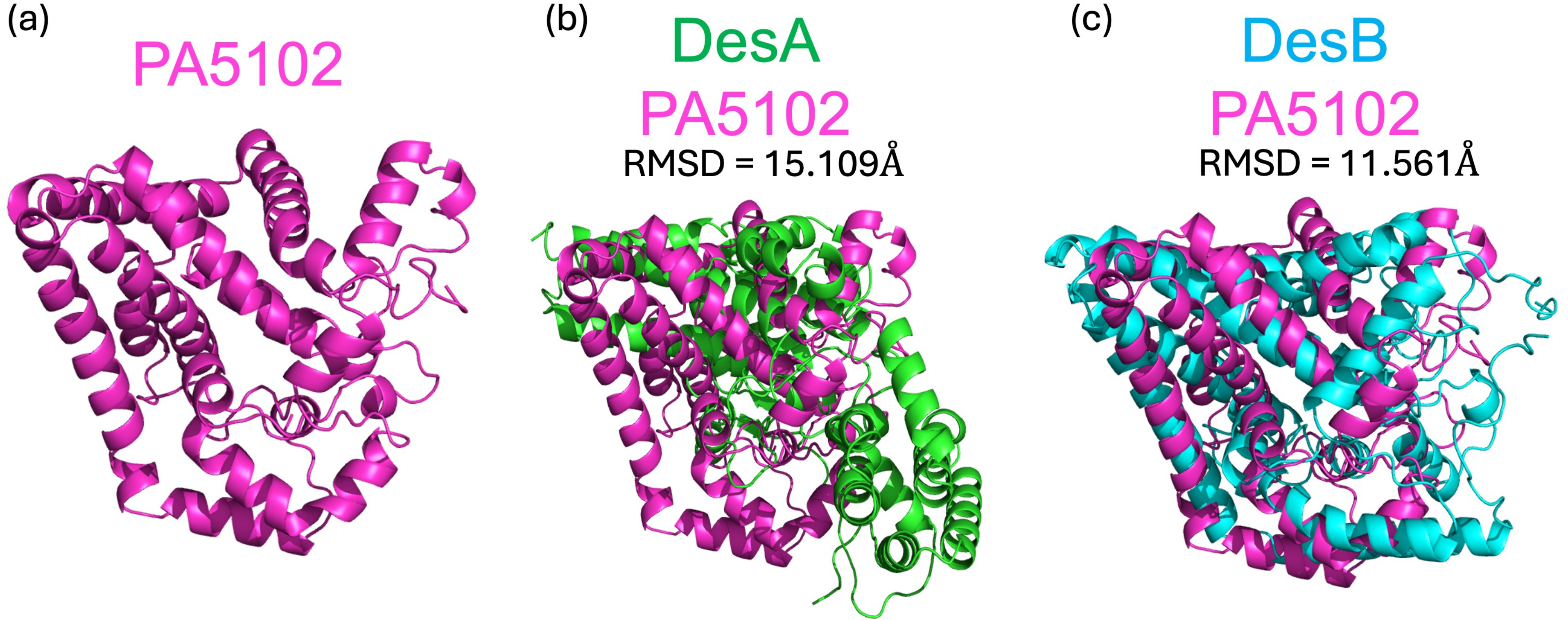

(a)
PA5102
(b)
DesA
PA5102
RMSD = 15.109Å
(c)
DesB
PA5102
RMSD = 11.561Å
